# Cardiac myosin-binding protein-C is a critical mediator of diastolic function

**DOI:** 10.1007/s00424-014-1442-1

**Published:** 2014-01-19

**Authors:** Carl W. Tong, Nandini A. Nair, Karen M. Doersch, Yang Liu, Paola C. Rosas

**Affiliations:** 1 Department of Medical Physiology, Texas A&M Health Science Center, 702 Southwest H.K. Dodgen Loop, Temple, TX 76504 USA; 2 Department of Medicine/Cardiology Division, Baylor Scott & White Healthcare, Temple, TX USA; 3 Advanced Heart Failure, Mechanical Circulatory Support, Heart Transplant Program, Providence Spokane Cardiology, Spokane, WA USA

**Keywords:** Cardiac myosin-binding protein-C, MyBPC3, Diastolic dysfunction, Heart failure with preserved ejection fraction, HFpEF

## Abstract

Diastolic dysfunction prominently contributes to heart failure with preserved ejection fraction (HFpEF). Owing partly to inadequate understanding, HFpEF does not have any effective treatments. Cardiac myosin-binding protein-C (cMyBP-C), a component of the thick filament of heart muscle that can modulate cross-bridge attachment/detachment cycling process by its phosphorylation status, appears to be involved in the diastolic dysfunction associated with HFpEF. In patients, cMyBP-C mutations are associated with diastolic dysfunction even in the absence of hypertrophy. cMyBP-C deletion mouse models recapitulate diastolic dysfunction despite in vitro evidence of uninhibited cross-bridge cycling. Reduced phosphorylation of cMyBP-C is also associated with diastolic dysfunction in patients. Mouse models of reduced cMyBP-C phosphorylation exhibit diastolic dysfunction while cMyBP-C phosphorylation mimetic mouse models show enhanced diastolic function. Thus, cMyBP-C phosphorylation mediates diastolic function. Experimental results of both cMyBP-C deletion and reduced cMyBP-C phosphorylation causing diastolic dysfunction suggest that cMyBP-C phosphorylation level modulates cross-bridge detachment rate in relation to ongoing attachment rate to mediate relaxation. Consequently, alteration in cMyBP-C regulation of cross-bridge detachment is a key mechanism that causes diastolic dysfunction. Regardless of the exact molecular mechanism, ample clinical and experimental data show that cMyBP-C is a critical mediator of diastolic function. Furthermore, targeting cMyBP-C phosphorylation holds potential as a future treatment for diastolic dysfunction.

## Background

Heart failure occurs when cardiac output cannot meet the body’s demand. It has an estimated global prevalence of 23 M [4]. Lifetime risks for developing heart failure of a 55-year-old European and a 40-year-old American are 30.2 and 20 %, respectively [2, 15]. Despite treatment advances, 5-year mortality of heart failure patients remains high at 42–80 % [49]. Heart failure can occur with left ventricular ejection fraction (EF) of ≥50 %, which is defined as heart failure with persevered ejection fraction (HFpEF) [29, 49]. Prevalence of HFpEF has increased to 47 % of all heart failure cases [36]. Diastolic dysfunction is the generally accepted cause of HFpEF [29]. Diastolic dysfunction also occurs with heart failure with reduced ejection fraction (HFrEF) [39], defined as EF < 40 % [49]. Hypertrophic cardiomyopathy (HCM) patients progress to heart failure with type distribution of 48 % HFpEF, 30 % HFrEF, and 22 % outflow obstruction [30]. HCM patients with primarily diastolic dysfunction and without outflow obstruction experience the shortest progression from HCM diagnosis to heart failure [30]. Mere diagnosis of mild diastolic dysfunction carries >eightfold increase in mortality over 5 years [39]. Unfortunately, pathogenic mechanisms that cause diastolic dysfunction remain enigmatic. With this perspective, this review summarizes evidence that cardiac myosin-binding protein-C mediates diastolic function.

**Fig. 1:**
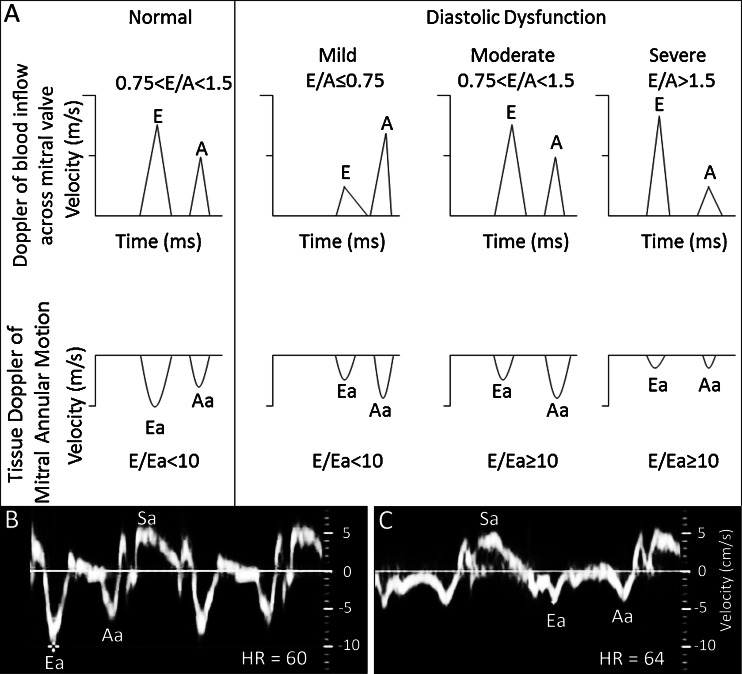
Doppler flow schematic and patient tissue Doppler example. **a** *E* is the peak blood flow Doppler across mitral valve during early diastolic filling. *A* is the peak blood flow Doppler across mitral valve during atrial contraction of diastole. E will initially decrease with mild diastolic function but increases with worsening diastolic dysfunction. The E/A ratio will initially decrease with mild diastolic dysfunction but increases with worsening diastolic dysfunction to make moderate–severe diastolic dysfunction indistinguishable from normal to enhanced diastolic function. *Ea* is the peak heart muscle relaxation TD during early diastole about mitral valve annulus. Ea monotonically decreases with worsening diastolic dysfunction. *Aa* is the peak heart muscle expansion TD during atrial contraction phase of diastole. *Sa* is the peak heart muscle contraction TD during systole. **b** TD of a normal 62-year-old male. **c** TD of 66-year-old male with severe diastolic dysfunction (note severely slowed Ea and reduced Ea/Sa). Time scales are different between (**b**) and (**c**)

To facilitate understanding, this paragraph summarizes echocardiographic Doppler measurements that are used to quantify in vivo diastolic function. Early diastolic (Ea) is the tissue Doppler (TD) measurement of the peak heart muscle relaxation velocity about mitral valve annulus during early diastole (Fig. 1). Ea is an extraordinarily reliable echocardiographic measurement of diastolic function because it correlates with diastolic hemodynamics indices (pressure decay time constant, peak pressure decay rate (−dP/dt)_min_, pressure/volume relationship during diastolic filling) and monotonically decreases with worsening diastolic dysfunction [20, 24, 32, 35, 39] (Fig. 1). Ea is also referred as e′, E′, or Em [32]. Systolic (Sa) is the TD of peak heart muscle contraction velocity during systole (Fig. 1). The Doppler of the peak blood flow velocity across the mitral valve during early diastole is named E [20, 24, 32, 39]. E initially decreases with mild diastolic dysfunction but increases with worsening diastolic dysfunction due to resultant left atrial dilation leading to increases in left atrial pressure [20, 32, 39]. Thus, increasing E/Ea ratio indicates worsening diastolic dysfunction by capturing both increasing left atrial pressure and myocardium’s decreasing ability to relax [20, 24, 32, 39] (Fig. 1).

## Need for cMyBP-C

Cardiac myosin-binding protein-C (cMyBP-C) is a part of the thick filament of the heart muscle [28]. Although cMyBP-C is believed to repress myosin–actin interaction by different mechanisms [12, 18], an important mechanism is that cMyBP-C binding to the rod region of myosin can slow cross-bridge detachment to impair relaxation [1, 12, 26]. Thus, cMyBP-C mutations may lead to diastolic dysfunction. Mutations in cMyBP-C are a leading cause of hypertrophic cardiomyopathy (HCM) [18]. HCM patients, a significant portion of whom carry cMyBP-C mutations, can present with diastolic dysfunction (demonstrated by slowed heart muscle relaxation velocity Ea) before the onset of hypertrophy [19, 33, 34]. A cohort of pediatric HCM patients, 19/27 of whom have cMyBP-C mutations, demonstrates diastolic dysfunction without hypertrophy [37]. Another cohort of patients with three common cMyBP-C mutations found in the Netherlands exhibits hypertrophy with diastolic dysfunction or prehypertrophy with TD evidence of impaired relaxation [31]. The presentation of diastolic dysfunction before the onset of hypertrophy suggests that cMyBP-C mutations cause diastolic dysfunction independent of hypertrophy. Furthermore, a single nucleotide polymorphism in cMyBP-C has been found in diastolic heart failure patients [48]. Thus, clinical evidence suggests that nonmutated/normal cMyBP-C is needed for normal diastolic function.

**Fig. 2:**
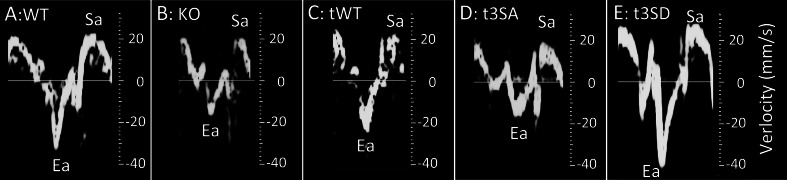
Mouse TD of myocardium at mitral valve annulus examples. **a** wild type, **b** cMyBP-C(-/-, Ex3-10), **c** cMyBP-C(tWT), **d** cMyBP-C(t3SA), and **e** cMyBP-C(t3SD). cMyBP-C(-/-. Ex3-10) and cMyBP-C(t3SA) show slowed Ea and reduced Ea/Sa

Animal models support the clinical finding that the loss of cMyBP-C causes diastolic dysfunction. Targeting exons 3–10, Harris et al. created the first cMyBP-C null (i.e., complete loss of cMyBP-C expression) mouse model cMyBP-C(-/-, Ex3-10) [17]. cMyBP-C(-/-, Ex3-10) hearts exhibit diastolic dysfunction with slowed Ea (Fig. 2a, b) and increased E/Ea ratio similar to human patients [44] with confirmatory intracardiac pressure measurements of slower (−dP/dt)_min_ and longer pressure decay constant τ [3]. Another cMyBP-C null mouse model, cMyBP-C(-/-, Ex1-2), which was made by targeting preexon-1 to exon-2, demonstrates impaired relaxation by slower (−dP/dt)_min_ and longer pressure decay constant τ [5]. Additionally, cMyBP-C mutation homozygous and heterozygous knock-in models exhibit diastolic dysfunction with elevated E/Ea ratio but faster intracellular calcium [Ca^2+^]_i_, demonstrating that impaired relaxation is caused by myofilament dysfunction, not by slowed calcium handling [13]. Furthermore, a conditional cMyBP-C knockout mouse model demonstrates diastolic dysfunction without hypertrophy after induction of the cMyBP-C deletion [6]. Thus, the presence of nonmutated cMyBP-C is required for normal diastolic function.

## Mediation of diastolic function by posttranslational modification of cMyBP-C

cMyBP-C phosphorylation levels have been found to be decreased by >50 % in explanted hearts from patients with end-stage heart failure during heart transplant [8, 11, 21, 25]. End-stage failing hearts have severe diastolic and systolic dysfunction along with calcium and metabolic derangements; therefore, it is difficult to assess the impact of cMyBP-C phosphorylation. Samples obtained during myomectomy surgery to relieve outflow obstruction showed that HCM hearts have decreased cMyBP-C phosphorylation levels [8, 10, 21]. HCM hearts exhibit predominantly diastolic dysfunction, implying that reduced cMyBP-C phosphorylation is an underlying cause.

Animal models suggest that cMyBP-C phosphorylation mediates diastolic function. Protein kinase A (PKA) can phosphorylate human cMyBP-C at S275, S284, and S304 [14] and their mouse equivalents (S273, S282, S302) as confirmed by mass spectrometry [23]. Expressing cMyBP-C with S273A, S282A, and S302A and S273D, S282D, and S302D mutations onto cMyBP-C(-/-, Ex3-10) background created cMyBP-C(t3SA) (phosphorylation deficient) [44] and cMyBP-C(t3SD) (phosphorylation mimetic) [7, 26] mouse models, respectively. These mouse models allow one to elucidate the impact of cMyBP-C phosphorylation at its known PKA sites. Myosin-binding protein C (cMyBP-C)(t3SA) hearts exhibited similar EF [7, 26, 44], reduced Ea (slowed heart muscle relaxation TD velocity, Fig. 2), and increased E/Ea ratio (diastolic dysfunction) [26, 44] in comparison to its wild-type equivalent cMyBP-C(tWT) control, suggesting that reduced cMyBP-C phosphorylation causes predominantly diastolic dysfunction. Furthermore, cMyBP-C(t3SA) mice resemble human HFpEF with shorter voluntary running distances, pulmonary edema, and elevated brain natriuretic peptide levels [26]. Another cMyBP-C phosphorylation-deficient mouse model cMyBP-C(t/t,AllP-) was made by expressing cMyBP-C with five mutations (T272A, S273A, T281A, S282A, S302A) onto the cMyBP-C truncation background of cMyBP-C(t/t) [41]. Unlike cMyBP-C(t3SA), cMyBP-C(t/t, AllP-) hearts showed ~50 % reduction in fractional shortening and severely dilated ventricles in comparison to its cMyBP-C(t/t, WT) control [41], suggesting that cMyBP-C phosphorylation also mediates systolic function. Differences in mutations and mouse backgrounds probably caused the different phenotypes in these two cMyBP-C phosphorylation-deficient mouse models. Subsequently, expressing combinatorial phosphorylation site mutations (S282A-SAS, S273A/S282D/S302A-ADA, and S273D/S282A/S302D-DAD) onto the cMyBP-C(t/t) background made mutant hearts that exhibit similar EF as their control cMyBP-C(t/t, WT), providing evidence that cMyBP-C phosphorylation has greater impact on diastolic function [40]. More recently, expressing phosphorylation-deficient cMyBP-C mutants of AAD(T272A,S273A,T281A,S282A,S302D) and DAA(T272D,S273D,T281A,S282A,S302A) onto cMyBP-C(t/t) background led to reduced EF and impaired relaxation as evidenced by slowed heart muscle relaxation TD velocity Ea [16]. Conversely, the phosphorylation-mimetic cMyBP-C(t3SD) demonstrated enhanced diastolic function by faster heart muscle relaxation TD velocity Ea (Fig. 2) and reduced E/Ea ratio (enhanced diastolic function) [26]. Together, these findings indicate that cMyBP-C phosphorylation mediates diastolic function.

Posttranslational modifications of cMyBP-C other than phosphorylation may also affect diastolic function. Unilateral nephrectomy and chronic deoxycorticosterone acetate (DOCA) salt treatment will cause diastolic dysfunction [27]. Diastolic dysfunction in this mouse model was attributed to altered myofilament calcium sensitivity due to increased glutathionylation of cMyBP-C [27]. Tetrahydrobiopterin treatment decreased glutathionylation and increased cross-bridge cycling rate to reverse diastolic dysfunction independent of cMyBP-C phosphorylation [22]. Thus, glutathionylation of cMyBP-C may also mediate diastolic dysfunction.

### Possible mechanism

**Fig. 3:**
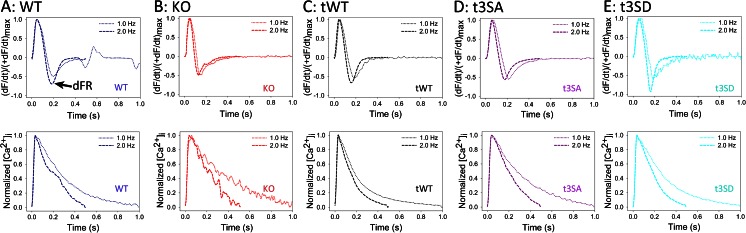
Papillary muscle experiment examples. *Top panels* show time course of dF/dt normalized to (dF/dt)_max_. *Bottom panels* show corresponding time course of normalized intracellular calcium concentrations. dFR = (+dF/dt)_max_/(−dF/dt)_min_. Increasing magnitude of dFR represents acceleration of relaxation. **a** wild type, **b** cMyBP-C(-/-, Ex3-10), **c** cMyBP-C(tWT), **d** cMyBP-C(t3SA), and **e** cMyBP-C(t3SD). cMyBP-C(-/-, Ex3-10) and cMyBP-C(t3SA) muscles exhibit smaller dFRs that do not change with increasing pacing frequency

**Fig. 4:**
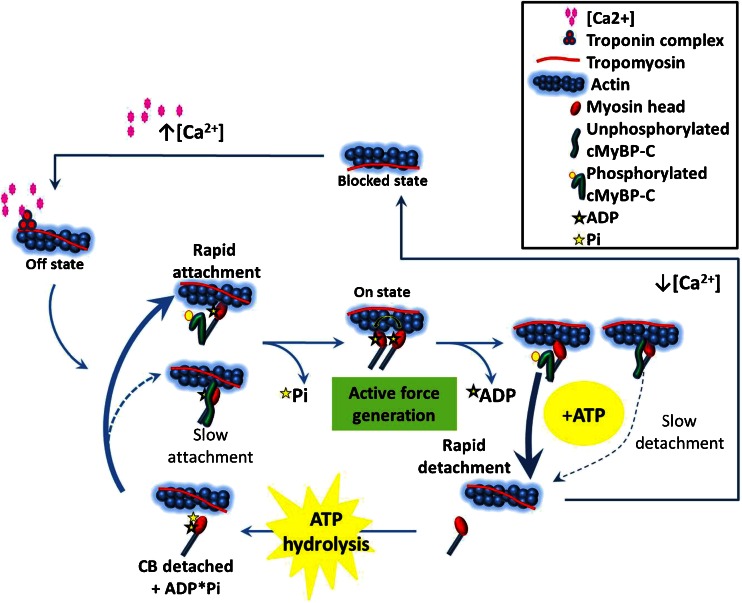
Hypothesis schematic. Increasing [Ca2+]_i_ moves tropomyosin from blocked to off state. Phosphorylated cMyBP-C facilitates rapid cross-bridge attachment. Transition of cross-bridges from weakly bound to strongly bound states with release of Pi causes further displacement of tropomyosin to fully activate thin filament to on state. Phosphorylated cMyBP-C accelerates cross-bridge detachment in reference to attachment. Thin filament free of attached cross-bridges can snap back into the blocked state with decreasing [Ca2+]_i_

cMyBP-C phosphorylation may mediate diastolic function by modulating the relative cross-bridge detachment rate with respect to cross-bridge attachment rate (Fig. 4). Myocardial stretch activation experiments [43, 44] and motility assays using native thick filament [38] demonstrate that both cMyBP-C phosphorylation and cMyBP-C deletion increase cross-bridge cycling rates. Surprisingly, cMyBP-C deletion causes diastolic dysfunction despite its constitutively fast cross-bridge cycling rates [16, 38, 44]. Correlating echocardiographic TD measurements (Ea, Sa) and intact papillary muscle results solves this paradox. cMyBP-C(-/-, Ex3-10) and cMyBP-C phosphorylation-deficient cMyBP-C(t3SA) hearts show characteristic slowed Ea and reduced Ea/Sa ratio (Fig. 2) [46, 47]. Ea and Sa correspond to (dP/dt)_min_ and (dP/dt)_max_, respectively [35, 42]. Since pressure is a function of force, then (dF/dt)_min_, (dF/dt)_max_, and derivative force ratio (dFR) = (dF/dt)_min_/(dF/dt)_max_ measured from intact papillary muscles are analogous to Ea, Sa, and Ea/Sa, respectively. cMyBP-C(-/-, Ex3-10) and cMyBP-C(t3SA) papillary muscles show decreased dFR, reflecting reduced Ea/Sa [45, 46]. Increasing dFR equates to acceleration of relaxation because peak relaxation rate (dF/dt)_min_ increases exceed increases in peak force generation rate (dF/dt)_max_. Increased pacing frequency increases dFR only in papillary muscles with phosphorylatable cMyBP-C (Fig. 3) [45–47]. Increased pacing frequency causes similar shortening of [Ca^2+^]_i_ decay times in all the mouse models (Fig. 3) [45–47]. Therefore, the accelerated relaxation can be attributed to phosphorylated cMyBP-C increasing cross-bridge detachment rate faster than attachment rate but not to changes in calcium handling. cMyBP-C(-/-, Ex3-10) lacks cMyBP-C to modulate cross-bridge detachment causing an inability to accelerate relaxation (slow and unchanging dFR in Fig. 3) despite its fast cross-bridge cycling, resulting in smaller Ea/Sa (Fig. 2). Similarly, cMyBP-C(t3SA) mutants are unable to increase relative cross-bridge detachment rate, causing depressed dFR (Figs. 3 and 4) and seen at the whole heart level by smaller Ea/Sa (Fig. 2). Furthermore, phosphorylated cMyBP-C has been shown to increase cross-bridge detachment rate without affecting attachment rate [9]. Together, these results combine to suggest that phosphorylated cMyBP-C modulates cross-bridge detachment rate in relation to attachment rate to mediate diastolic function.

### Conclusion

Clinical evidence and animal models demonstrate that cMyBP-C mediates diastolic function. The correlation of intact papillary muscle experiments and in vivo TD measurements suggests that cMyBP-C phosphorylation modulates relative cross-bridge detachment rate with respect to attachment rate to mediate diastolic function. Thus, targeting cMyBP-C phosphorylation holds great potential for the treatment of diastolic dysfunction.
